# (2-Formyl-6-methoxy­phenolato-κ^2^ *O* ^1^,*O* ^2^)(perchlorato-κ*O*)(1,10-phenanthroline-κ^2^ *N*,*N*′)copper(II)

**DOI:** 10.1107/S1600536808009975

**Published:** 2008-04-16

**Authors:** Feng-Ying Dong, Yun-Ming Sun, Yan-Tuan Li, Zhi-Yong Wu

**Affiliations:** a Marine Drug and Food Institute, Ocean University of China, Qingdao 266003, People’s Republic of China; b Department of Plant Protection, Qingdao Agricultural University, Qingdao 266109, People’s Republic of China

## Abstract

In the title mol­ecule, [Cu(C_8_H_7_O_3_)(ClO_4_)(C_12_H_8_N_2_)], the Cu^II^ ion is five-coordinated by two N atoms [Cu—N = 1.995 (3) and 2.022 (3) Å] from a 1,10-phenanthroline ligand, two O atoms [Cu—O = 1.908 (2) and 1.927 (2) Å] from an *o*-vanillin ligand and one O atom [Cu—O = 2.510 (3) Å] from a perchlorate anion in a distorted square-pyramidal geometry. Three O atoms of the perchlorate anion are rotationally disordered between two orientations, with occupancies of 0.525 (13) and 0.475 (13). In the crystal structure, two mol­ecules related by a centre of symmetry are paired in such a way that the phenolate O atom from one mol­ecule completes the distorted octa­hedral Cu coordination in another mol­ecule [Cu⋯O = 2.704 (2) Å].

## Related literature

For general background, see: Janzen *et al.* (2004 ▶). For related structures, see: Plieger *et al.* (2004 ▶); Lin & Zeng (2006 ▶); Youngme *et al.* (2005 ▶).
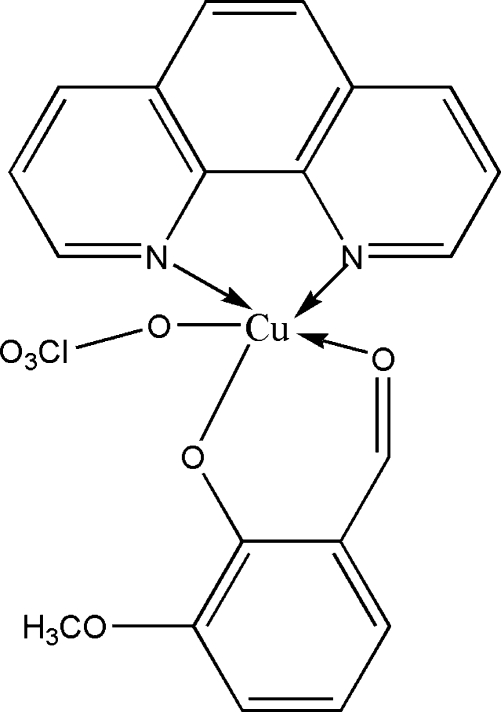


## Experimental

### 

#### Crystal data

[Cu(C_8_H_7_O_3_)(ClO_4_)(C_12_H_8_N_2_)]
*M* *_r_* = 494.33
Monoclinic, 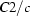

*a* = 22.332 (2) Å
*b* = 9.3986 (9) Å
*c* = 18.339 (2) Å
β = 96.733 (2)°
*V* = 3822.7 (7) Å^3^
*Z* = 8
Mo *K*α radiation
μ = 1.33 mm^−1^
*T* = 298 (2) K
0.26 × 0.17 × 0.13 mm

#### Data collection

Bruker SMART CCD area-detector diffractometer
Absorption correction: multi-scan (*SADABS*; Sheldrick, 1996 ▶) *T*_min_ = 0.723, *T*_max_ = 0.846
9561 measured reflections
3374 independent reflections
2731 reflections with *I* > 2σ(*I*)
*R*_int_ = 0.026

#### Refinement

*R*[*F* ^2^ > 2σ(*F* ^2^)] = 0.040
*wR*(*F* ^2^) = 0.109
*S* = 1.00
3374 reflections
309 parameters
H-atom parameters constrained
Δρ_max_ = 0.52 e Å^−3^
Δρ_min_ = −0.53 e Å^−3^

### 

Data collection: *SMART* (Bruker, 1998 ▶); cell refinement: *SAINT* (Bruker, 1998 ▶); data reduction: *SAINT*; program(s) used to solve structure: *SHELXS97* (Sheldrick, 2008 ▶); program(s) used to refine structure: *SHELXL97* (Sheldrick, 2008 ▶); molecular graphics: *SHELXTL* (Sheldrick, 2008 ▶); software used to prepare material for publication: *SHELXTL*.

## Supplementary Material

Crystal structure: contains datablocks global, I. DOI: 10.1107/S1600536808009975/cv2393sup1.cif

Structure factors: contains datablocks I. DOI: 10.1107/S1600536808009975/cv2393Isup2.hkl

Additional supplementary materials:  crystallographic information; 3D view; checkCIF report

## Figures and Tables

**Fig. 1.**
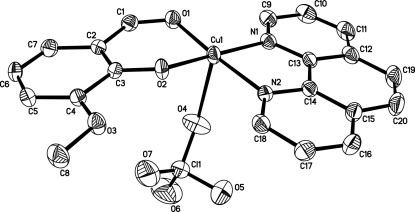
The molecular structure of (I) showing the atom-numbering scheme and 30% probability displacement ellipsoids. Only major part of the disordered perchlorate anion is drawn. H atoms omitted for clarity.

**Fig. 2.**
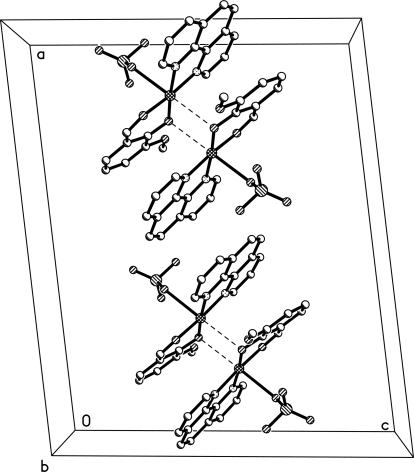
A portion of the crystal packing showing the paired molecules with the short intermolecular Cu···O distances (dashed lines) of 2.704 (2) Å. For disordered perchlorate anions, only major parts are drawn. H atoms omitted for clarity.

## supplementary crystallographic information

### Comment

Studies of complexes containing salicylaldehyde and its derivatives have been reported by Janzen *et al.* (2004) and other groups. The five-coordinated Cu^II^ complexes have been extensively investigated and the relationship between their structures and reactivities is of major importance. We report here the synthesis and structure of the title complex, (I).

In complex (I), the Cu^II^ ion is five-coordinated by N1 and N2 atoms from 1,10-phenanthroline ligand, O1 and O2 atoms from *o*-vanillin and O4 atom from perchlorate anion in a distorted square-pyramidal geometry. Atom O4 lies in the axial position and the equatorial positions are occupied by the other four donor atoms. The bond distances for Cu1—N1 and Cu1—N2 of 2.022 (3)Å and 1.995 (3)Å, respectively, are nearly as long as those found for the similar auxiliary phen ligand (Youngme *et al.*, 2005). The bond lengths for Cu1—O1 and Cu1—O2 of 1.927 (2)Å and 1.908 (2) Å, respectively, are slightly shorter than those of reported *o*-vanillin complex [1.965 (2) and 1.9201 (17) Å; Lin & Zeng, 2006]. The Cu1—O4 bond distance of 2.510 (3)Å is in the range observed in analogous compound [2.381 (4)Å and 2.559 (4) Å; Plieger *et al.*, 2004]. The larger angles around Cu are near 180°, so the ligands form a satisfactory N_2_ O_2_ square, with atom O4 inhabiting the axial position. In this way, a distorted square-pyramid is formed (Fig. 1). Three O atoms of perchlorate anion are rotationally disordered between two orientations with the occupancies of 0.525 (13) and 0.475 (13), respectively.

In the crystal, two molecules related by centre of symmetry are paired in such a way, that phenolate O atom from one molecule complete the distorted octahedral Cu coordination [Cu—O 2.704 (2) Å] in another molecule (Fig. 2).

### Experimental

To a solution of Cu(ClO_4_)_2_^.^6H_2_O (2 mmol, 741 mg) in water (25 ml) was added the mixture of 1,10-phenanthroline (2 mmol, 396 mg), *o*-vanillin (2 mmol, 304 mg) and NaOH (2 mmol, 40 mg) in ethanol (30 ml) and water (10 ml). The resulting solution was refluxed for 3 h and then concentrated to 40 ml. On standing for a week at room temperature complex (I) formed as green crystals. The crystals were isolated, washed three times with methanol and dried in a vacuum desiccator using anhydrous CaCl_2_ (yield 86%). Analysis; calculated for C_20_H_15_ClN_2_O_7_Cu: C, 48.59; H, 3.06; N, 5.67%; Found: C, 48.61; H, 3.08; N, 5.64%.

### Refinement

H atoms were positioned geometrically [0.93 (CH) and 0.96 (CH_3_) Å] and constrained to ride on their parent atoms with *U*_iso_(H) = 1.2 (1.5 for methyl) *U*_eq_. Atoms of O5, O6 and O7 appeared to be disordered, and were refined as two parts (occupancy factors are 0.525 (13) and 0.475 (13), respectively).

### Crystal data

**Table:**

[Cu(C_8_H_7_O_3_)(ClO_4_)(C_12_H_8_N_2_)]	*F*_000_ = 2008
*M**_r_* = 494.33	*D*_x_ = 1.718 Mg m^−^^3^
Monoclinic, *C*2/*c*	Mo *K*α radiation λ = 0.71073 Å
Hall symbol: -C 2yc	Cell parameters from 3881 reflections
*a* = 22.332 (2) Å	θ = 2.2–27.3º
*b* = 9.3986 (9) Å	µ = 1.33 mm^−^^1^
*c* = 18.339 (2) Å	*T* = 298 (2) K
β = 96.733 (2)º	Block, green
*V* = 3822.7 (7) Å^3^	0.26 × 0.17 × 0.13 mm
*Z* = 8	

### Data collection

**Table:**

Bruker SMART CCD area-detector diffractometer	3374 independent reflections
Radiation source: fine-focus sealed tube	2731 reflections with *I* > 2σ(*I*)
Monochromator: graphite	*R*_int_ = 0.026
*T* = 298(2) K	θ_max_ = 25.0º
φ and ω scans	θ_min_ = 1.8º
Absorption correction: multi-scan(SADABS; Sheldrick, 1996)	*h* = −26→22
*T*_min_ = 0.723, *T*_max_ = 0.846	*k* = −9→11
9561 measured reflections	*l* = −21→21

### Refinement

**Table:**

Refinement on *F*^2^	Secondary atom site location: difference Fourier map
Least-squares matrix: full	Hydrogen site location: inferred from neighbouring sites
*R*[*F*^2^ > 2σ(*F*^2^)] = 0.040	H-atom parameters constrained
*wR*(*F*^2^) = 0.109	*w* = 1/[σ^2^(*F*_o_^2^) + (0.0555*P*)^2^ + 9.3448*P*] where *P* = (*F*_o_^2^ + 2*F*_c_^2^)/3
*S* = 1.01	(Δ/σ)_max_ = 0.001
3374 reflections	Δρ_max_ = 0.52 e Å^−^^3^
309 parameters	Δρ_min_ = −0.53 e Å^−^^3^
Primary atom site location: structure-invariant direct methods	Extinction correction: none

### Special details

**Table:**

Geometry. All e.s.d.'s (except the e.s.d. in the dihedral angle between two l.s. planes) are estimated using the full covariance matrix. The cell e.s.d.'s are taken into account individually in the estimation of e.s.d.'s in distances, angles and torsion angles; correlations between e.s.d.'s in cell parameters are only used when they are defined by crystal symmetry. An approximate (isotropic) treatment of cell e.s.d.'s is used for estimating e.s.d.'s involving l.s. planes.
Refinement. Refinement of *F*^2^ against ALL reflections. The weighted *R*-factor *wR* and goodness of fit *S* are based on *F*^2^, conventional *R*-factors *R* are based on *F*, with *F* set to zero for negative *F*^2^. The threshold expression of *F*^2^ > σ(*F*^2^) is used only for calculating *R*-factors(gt) *etc*. and is not relevant to the choice of reflections for refinement. *R*-factors based on *F*^2^ are statistically about twice as large as those based on *F*, and *R*- factors based on ALL data will be even larger.

### Fractional atomic coordinates and isotropic or equivalent isotropic displacement parameters (Å^2^)

**Table:**

	*x*	*y*	*z*	*U*_iso_*/*U*_eq_	Occ. (<1)
Cu1	0.188504 (18)	0.31643 (4)	0.04538 (2)	0.03647 (16)	
Cl1	0.10243 (5)	0.11761 (11)	0.16636 (6)	0.0566 (3)	
N1	0.13385 (12)	0.4848 (3)	0.01905 (15)	0.0371 (6)	
N2	0.13152 (12)	0.2220 (3)	−0.03161 (14)	0.0335 (6)	
O1	0.23634 (11)	0.4265 (2)	0.11935 (13)	0.0423 (6)	
O2	0.23876 (10)	0.1526 (2)	0.06151 (12)	0.0384 (6)	
O3	0.27110 (11)	−0.1131 (2)	0.08481 (13)	0.0454 (6)	
O4	0.12199 (17)	0.2487 (3)	0.14103 (19)	0.0820 (10)	
O5	0.0588 (4)	0.0750 (10)	0.1043 (5)	0.109 (4)	0.525 (13)
O6	0.0777 (7)	0.1165 (15)	0.2296 (6)	0.133 (6)	0.525 (13)
O7	0.1471 (4)	0.0082 (10)	0.1615 (7)	0.115 (5)	0.525 (13)
O5'	0.1432 (5)	0.1020 (11)	0.2320 (6)	0.116 (5)	0.475 (13)
O6'	0.1046 (7)	0.0038 (12)	0.1247 (6)	0.125 (5)	0.475 (13)
O7'	0.0456 (5)	0.1427 (13)	0.1921 (8)	0.107 (5)	0.475 (13)
C1	0.27125 (15)	0.3732 (4)	0.17002 (19)	0.0397 (8)	
H1	0.2873	0.4352	0.2068	0.048*	
C2	0.28934 (14)	0.2287 (4)	0.17814 (18)	0.0343 (7)	
C3	0.27120 (14)	0.1246 (4)	0.12378 (17)	0.0327 (7)	
C4	0.29105 (15)	−0.0172 (4)	0.13831 (17)	0.0355 (7)	
C5	0.32785 (15)	−0.0494 (4)	0.20140 (19)	0.0427 (8)	
H5	0.3408	−0.1427	0.2098	0.051*	
C6	0.34618 (16)	0.0553 (4)	0.2532 (2)	0.0471 (9)	
H6	0.3714	0.0314	0.2954	0.057*	
C7	0.32762 (16)	0.1911 (4)	0.24245 (19)	0.0446 (9)	
H7	0.3400	0.2601	0.2773	0.054*	
C8	0.2885 (2)	−0.2566 (4)	0.0982 (2)	0.0574 (11)	
H8A	0.3317	−0.2631	0.1047	0.086*	
H8B	0.2727	−0.3143	0.0571	0.086*	
H8C	0.2729	−0.2898	0.1417	0.086*	
C9	0.13505 (17)	0.6141 (4)	0.0476 (2)	0.0461 (9)	
H9	0.1648	0.6364	0.0857	0.055*	
C10	0.09285 (19)	0.7191 (4)	0.0221 (2)	0.0562 (11)	
H10	0.0945	0.8088	0.0436	0.067*	
C11	0.04975 (18)	0.6887 (4)	−0.0340 (2)	0.0554 (11)	
H11	0.0217	0.7577	−0.0511	0.067*	
C12	0.04740 (16)	0.5535 (4)	−0.0665 (2)	0.0470 (9)	
C13	0.09052 (14)	0.4546 (4)	−0.03700 (18)	0.0362 (8)	
C14	0.08937 (15)	0.3117 (4)	−0.06422 (18)	0.0360 (8)	
C15	0.04546 (16)	0.2703 (4)	−0.12121 (19)	0.0451 (9)	
C16	0.04672 (17)	0.1275 (5)	−0.1436 (2)	0.0528 (10)	
H16	0.0186	0.0944	−0.1811	0.063*	
C17	0.08918 (18)	0.0381 (4)	−0.1103 (2)	0.0505 (9)	
H17	0.0903	−0.0563	−0.1253	0.061*	
C18	0.13112 (16)	0.0874 (4)	−0.05372 (19)	0.0420 (8)	
H18	0.1595	0.0245	−0.0309	0.050*	
C19	0.00371 (17)	0.5082 (5)	−0.1256 (2)	0.0576 (11)	
H19	−0.0246	0.5732	−0.1468	0.069*	
C20	0.00277 (18)	0.3747 (5)	−0.1509 (2)	0.0582 (11)	
H20	−0.0265	0.3489	−0.1890	0.070*	

### Atomic displacement parameters (Å^2^)

**Table:**

	*U*^11^	*U*^22^	*U*^33^	*U*^12^	*U*^13^	*U*^23^
Cu1	0.0394 (3)	0.0299 (2)	0.0376 (2)	0.00616 (17)	−0.00553 (17)	0.00138 (18)
Cl1	0.0526 (6)	0.0501 (6)	0.0662 (7)	−0.0052 (5)	0.0029 (5)	0.0124 (5)
N1	0.0390 (15)	0.0334 (16)	0.0393 (15)	0.0047 (12)	0.0065 (13)	0.0045 (13)
N2	0.0315 (14)	0.0320 (15)	0.0366 (15)	0.0019 (11)	0.0017 (12)	0.0044 (12)
O1	0.0485 (14)	0.0300 (13)	0.0458 (14)	0.0028 (10)	−0.0058 (12)	−0.0011 (11)
O2	0.0459 (14)	0.0338 (13)	0.0321 (12)	0.0084 (10)	−0.0096 (10)	0.0003 (10)
O3	0.0582 (16)	0.0298 (13)	0.0453 (14)	0.0069 (11)	−0.0066 (12)	−0.0024 (11)
O4	0.116 (3)	0.054 (2)	0.083 (2)	−0.0084 (19)	0.041 (2)	0.0074 (18)
O5	0.086 (6)	0.104 (7)	0.124 (7)	−0.020 (5)	−0.037 (5)	0.023 (5)
O6	0.161 (17)	0.153 (11)	0.092 (8)	−0.031 (10)	0.047 (8)	0.032 (7)
O7	0.093 (7)	0.081 (6)	0.167 (12)	0.014 (5)	−0.003 (6)	0.038 (7)
O5'	0.134 (10)	0.098 (7)	0.099 (8)	0.017 (6)	−0.061 (6)	0.027 (6)
O6'	0.165 (16)	0.085 (7)	0.120 (9)	−0.016 (9)	0.001 (9)	−0.035 (7)
O7'	0.072 (7)	0.105 (7)	0.149 (13)	0.001 (5)	0.032 (6)	0.031 (8)
C1	0.0415 (19)	0.039 (2)	0.0375 (19)	−0.0060 (16)	0.0003 (16)	−0.0054 (16)
C2	0.0345 (17)	0.0327 (18)	0.0351 (17)	−0.0034 (14)	0.0017 (14)	0.0042 (14)
C3	0.0284 (16)	0.0364 (18)	0.0325 (17)	0.0007 (13)	−0.0002 (13)	0.0021 (15)
C4	0.0354 (17)	0.0359 (19)	0.0350 (18)	0.0014 (14)	0.0032 (14)	0.0034 (15)
C5	0.042 (2)	0.040 (2)	0.045 (2)	0.0049 (16)	0.0004 (16)	0.0116 (17)
C6	0.042 (2)	0.057 (2)	0.0379 (19)	−0.0005 (18)	−0.0116 (16)	0.0134 (18)
C7	0.046 (2)	0.050 (2)	0.0356 (19)	−0.0080 (17)	−0.0036 (16)	0.0003 (16)
C8	0.069 (3)	0.033 (2)	0.068 (3)	0.0084 (19)	0.000 (2)	0.001 (2)
C9	0.052 (2)	0.039 (2)	0.048 (2)	0.0036 (17)	0.0075 (17)	0.0036 (17)
C10	0.067 (3)	0.036 (2)	0.068 (3)	0.0133 (19)	0.020 (2)	0.004 (2)
C11	0.051 (2)	0.046 (2)	0.071 (3)	0.0207 (18)	0.012 (2)	0.019 (2)
C12	0.040 (2)	0.050 (2)	0.053 (2)	0.0131 (17)	0.0121 (17)	0.0177 (19)
C13	0.0325 (17)	0.041 (2)	0.0352 (17)	0.0056 (14)	0.0064 (14)	0.0131 (15)
C14	0.0327 (17)	0.041 (2)	0.0346 (17)	0.0029 (14)	0.0062 (14)	0.0078 (15)
C15	0.0359 (19)	0.057 (2)	0.041 (2)	−0.0003 (17)	0.0000 (15)	0.0052 (18)
C16	0.046 (2)	0.063 (3)	0.046 (2)	−0.0093 (19)	−0.0073 (18)	−0.002 (2)
C17	0.055 (2)	0.046 (2)	0.050 (2)	−0.0061 (18)	0.0014 (18)	−0.0046 (18)
C18	0.046 (2)	0.037 (2)	0.043 (2)	0.0028 (16)	0.0035 (16)	0.0057 (16)
C19	0.040 (2)	0.071 (3)	0.060 (3)	0.019 (2)	−0.0037 (18)	0.018 (2)
C20	0.040 (2)	0.078 (3)	0.053 (2)	0.007 (2)	−0.0105 (18)	0.007 (2)

### Geometric parameters (Å, °)

**Table:**

Cu1—O2	1.908 (2)	C5—H5	0.9300
Cu1—O1	1.927 (2)	C6—C7	1.349 (5)
Cu1—N2	1.995 (3)	C6—H6	0.9300
Cu1—N1	2.022 (3)	C7—H7	0.9300
Cu1—O4	2.510 (3)	C8—H8A	0.9600
Cl1—O6'	1.319 (10)	C8—H8B	0.9600
Cl1—O6	1.342 (11)	C8—H8C	0.9600
Cl1—O4	1.405 (3)	C9—C10	1.406 (5)
Cl1—O7'	1.424 (11)	C9—H9	0.9300
Cl1—O5'	1.429 (8)	C10—C11	1.355 (6)
Cl1—O7	1.442 (9)	C10—H10	0.9300
Cl1—O5	1.465 (8)	C11—C12	1.402 (6)
N1—C9	1.322 (5)	C11—H11	0.9300
N1—C13	1.357 (4)	C12—C13	1.401 (5)
N2—C18	1.329 (4)	C12—C19	1.436 (6)
N2—C14	1.350 (4)	C13—C14	1.432 (5)
O1—C1	1.246 (4)	C14—C15	1.402 (5)
O2—C3	1.305 (4)	C15—C16	1.404 (6)
O3—C4	1.368 (4)	C15—C20	1.431 (5)
O3—C8	1.417 (4)	C16—C17	1.358 (5)
C1—C2	1.419 (5)	C16—H16	0.9300
C1—H1	0.9300	C17—C18	1.393 (5)
C2—C7	1.418 (5)	C17—H17	0.9300
C2—C3	1.422 (5)	C18—H18	0.9300
C3—C4	1.420 (5)	C19—C20	1.337 (6)
C4—C5	1.372 (5)	C19—H19	0.9300
C5—C6	1.395 (5)	C20—H20	0.9300
			
O2—Cu1—O1	93.25 (9)	C5—C4—C3	120.6 (3)
O2—Cu1—N2	93.76 (10)	C4—C5—C6	121.1 (3)
O1—Cu1—N2	172.89 (10)	C4—C5—H5	119.4
O2—Cu1—N1	175.03 (10)	C6—C5—H5	119.4
O1—Cu1—N1	90.98 (11)	C7—C6—C5	120.5 (3)
N2—Cu1—N1	82.08 (11)	C7—C6—H6	119.7
O2—Cu1—O4	94.16 (11)	C5—C6—H6	119.7
O1—Cu1—O4	88.20 (11)	C6—C7—C2	120.1 (3)
N2—Cu1—O4	90.07 (12)	C6—C7—H7	119.9
N1—Cu1—O4	88.58 (11)	C2—C7—H7	119.9
O6'—Cl1—O6	122.8 (8)	O3—C8—H8A	109.5
O6'—Cl1—O4	119.1 (5)	O3—C8—H8B	109.5
O6—Cl1—O4	117.9 (6)	H8A—C8—H8B	109.5
O6'—Cl1—O7'	114.9 (8)	O3—C8—H8C	109.5
O6—Cl1—O7'	40.6 (6)	H8A—C8—H8C	109.5
O4—Cl1—O7'	106.8 (5)	H8B—C8—H8C	109.5
O6'—Cl1—O5'	110.0 (7)	N1—C9—C10	122.1 (4)
O6—Cl1—O5'	63.8 (8)	N1—C9—H9	118.9
O4—Cl1—O5'	100.1 (5)	C10—C9—H9	118.9
O7'—Cl1—O5'	104.0 (8)	C11—C10—C9	119.6 (4)
O6'—Cl1—O7	46.6 (6)	C11—C10—H10	120.2
O6—Cl1—O7	114.0 (8)	C9—C10—H10	120.2
O4—Cl1—O7	111.2 (4)	C10—C11—C12	120.1 (3)
O7'—Cl1—O7	141.9 (6)	C10—C11—H11	119.9
O5'—Cl1—O7	66.6 (6)	C12—C11—H11	119.9
O6'—Cl1—O5	52.7 (6)	C13—C12—C11	116.6 (4)
O6—Cl1—O5	111.5 (7)	C13—C12—C19	118.0 (4)
O4—Cl1—O5	100.8 (4)	C11—C12—C19	125.3 (4)
O7'—Cl1—O5	76.5 (7)	N1—C13—C12	123.4 (3)
O5'—Cl1—O5	157.9 (5)	N1—C13—C14	116.3 (3)
O7—Cl1—O5	99.1 (7)	C12—C13—C14	120.3 (3)
C9—N1—C13	118.2 (3)	N2—C14—C15	123.2 (3)
C9—N1—Cu1	129.9 (3)	N2—C14—C13	116.6 (3)
C13—N1—Cu1	111.9 (2)	C15—C14—C13	120.2 (3)
C18—N2—C14	118.7 (3)	C14—C15—C16	116.6 (3)
C18—N2—Cu1	128.4 (2)	C14—C15—C20	118.1 (4)
C14—N2—Cu1	112.9 (2)	C16—C15—C20	125.4 (4)
C1—O1—Cu1	123.8 (2)	C17—C16—C15	119.8 (3)
C3—O2—Cu1	123.7 (2)	C17—C16—H16	120.1
C4—O3—C8	116.3 (3)	C15—C16—H16	120.1
Cl1—O4—Cu1	133.3 (2)	C16—C17—C18	120.2 (4)
O1—C1—C2	127.5 (3)	C16—C17—H17	119.9
O1—C1—H1	116.3	C18—C17—H17	119.9
C2—C1—H1	116.3	N2—C18—C17	121.6 (3)
C7—C2—C1	117.5 (3)	N2—C18—H18	119.2
C7—C2—C3	120.4 (3)	C17—C18—H18	119.2
C1—C2—C3	122.1 (3)	C20—C19—C12	121.5 (4)
O2—C3—C4	118.8 (3)	C20—C19—H19	119.3
O2—C3—C2	123.9 (3)	C12—C19—H19	119.3
C4—C3—C2	117.2 (3)	C19—C20—C15	121.9 (4)
O3—C4—C5	124.9 (3)	C19—C20—H20	119.0
O3—C4—C3	114.6 (3)	C15—C20—H20	119.0
